# Practical Diagnosis

**Published:** 1897-01

**Authors:** 


PRACTICAL DIAGNOSIS. The Use of Symptoms in the Diagnosis of Disease. By HOBART AMORY HARE, M. D., Professor of Therapeutics and Materia Medica in the Jefferson Medical College of Philadelphia; Laureate of the Medical Society of London, of the Royal Academy in Belgium, etc. In one octavo volume of 566 pages, with 191 engravings and thirteen full-page colored plates. Philadelphia and New York: Lea Brothers & Co. 1896.

The author has seen fit in this work on diagnosis to depart radically from the old method of reaching a conclusion by wisely beginning at the bottom and building upward. Other treatises begin at the top and travel to the bottom, or, in other words, make the disease conform to the symptoms. Here the symptoms are studied with a view of reaching a diagnosis, and their application to determine the character of the disease follows. The treatise must necessarily be a very practical one, just such an one as a majority of physicians find they must consult. Especially in these days of new diseases, to which proper names have been applied, has a volume of this kind its greatest worth.

The chapters on the various symptoms of the diseases of the nervous system are unusually complete and interesting, and cannot fail to materially assist the physician who is somewhat unacquainted with these difficult affections. Many of the tables and illustrations in these chapters are taken from Dercum's system of nervous diseases, and are hence the latest and best information obtainable on these points. The chapter on the tongue is a valuable one, and the author belongs to that class who affirm that the diagnostic value of the tongue's appearance is worthy of study and consideration. Accompanying this chapter is a well-executed plate on the appearance of the tongue in typhoid fever, gastric catarrh and mucous disease.

The chapter on the general eye symptoms is a most important one, as these symptoms are generally noticed very early in the course of a disease and are very significant. Then follow chapters on the skin, the thorax and viscera, the abdomen and abdominal viscera, the blood-vessels and pulse, the blood, the urinary bladder and urine, and the bowels and feces.

Part II. is devoted to the manifestation of disease by symptoms and contains chapters on fever and subnormal temperatures, headache and vertigo, coma or unconsciousness, convulsions or general spasms, vomiting, regurgitation and the character of the vomit, cough and expectoration, pain, tendon reflexes and muscle tone and speech. The chapter on the blood is a very valuable exposition of one of the latest methods of diagnosis and is unusually complete. Special attention is paid to the appearance of the blood in leukemia, the malarial organisms in the blood and the filaria. Three beautiful colored plates accompany this chapter and help greatly to elucidate the text. A prominent feature of the book, present in nearly every chapter, is tables of symptoms differentiating closely allied diseases from a clinical standpoint, but which are pathologically widely different.

On the whole, the work is a very valuable one to the busy practitioner, but, like all good things, should not be abused; that is, he should not stop with a study of the symptoms merely, but go further and ascertain the causes and the pathology of the disease in question. In conclusion we quote from the letter of transmission:

"The two indexes form an especially valuable and practical portion of the work. In the index of diseases under each heading will be found annotated references to the various symptoms which constitute its clinical picture. Conversely, the index of symptoms, organs and terms furnishes a ready-reference list of the various diseases in which any given symptom may appear as a feature. It would be difficult to conceive of a work of greater utility. In connection with it the same author's Practical Therapeutics may be most advantageously consulted for the most approved treatment."

